# Is Adolescents’ Food Intake Associated with Exposure to the Food Intake of Their Mothers and Best Friends?

**DOI:** 10.3390/nu12030786

**Published:** 2020-03-17

**Authors:** Nina van den Broek, Junilla. K. Larsen, Maaike Verhagen, William J. Burk, Jacqueline M. Vink

**Affiliations:** Behavioural Science Institute, Radboud University; P.O. Box 9104, 6500 HE Nijmegen, The Netherlands; j.larsen@bsi.ru.nl (J.K.L.); m.verhagen@bsi.ru.nl (M.V.); w.burk@bsi.ru.nl (W.J.B.); j.vink@bsi.ru.nl (J.M.V.)

**Keywords:** food intake, snacking, adolescents, parents, peers

## Abstract

Both mothers’ and best friends’ food intake are associated with adolescents’ food intake, but they are rarely investigated simultaneously. In this study, we tested the associations of mothers’ and best friends’ food intake with adolescents’ intake of unhealthy and healthy food, obtained from home and from outside the home, and the moderating role of adolescents’ exposure to their food intake. Participants included 667 adolescents (53% female, *M*_age_ = 12.9) and 396 of their mothers. Within this adolescent sample, 378 best friend dyads were identified. All participants completed food frequency questionnaires. Mothers separately reported on their food intake in the presence and absence of their child, and adolescents indicated how often they ate and drank together with their best friend during school breaks. Mothers’, but not best friends’, food intake was positively related to adolescents’ intake of unhealthy and healthy food obtained from home and healthy food obtained from outside the home. Exposure to mothers’ healthy food intake magnified mother-child similarities in healthy food intake. Exposure to best friends’ intake of unhealthy food moderated adolescent-friend similarities in unhealthy food intake. Future work should assess the mechanisms that underlie these similarities, and should investigate these associations over time and in later developmental periods.

## 1. Introduction

Healthy eating during adolescence is critical to foster healthy physical and emotional growth, and to forestall the development of health-comprising conditions, such as obesity [1]. However, nowadays, many adolescents consume too much unhealthy food and too little healthy food. For example, sugar-sweetened beverages (SSBs) and sweet and savory snacks are found to contribute substantially to adolescents’ total caloric intake [2,3,4]. Conversely, it has been reported that most adolescents fail to meet the recommended intake of fruit and vegetables [5,6]. As a wealth of research has shown that the social environment shapes adolescents’ unhealthy and healthy food intake [7,8] and that consumption patterns established in adolescence tend to continue into adulthood [9,10], it is critical for prevention efforts to understand which social factors determine food intake during adolescence. Therefore, we aimed to investigate the role of (exposure to) the food intake of important socialization agents in explaining adolescents’ food intake.

A growing body of research highlights the important roles that both mothers and best friends play in explaining adolescents’ food intake. As a primary source of socialization, parents are often considered to be most influential in shaping their children’s food intake [11,12]. Although more attention has been given to the role of fathers in recent years [13], mothers are still considered to be the primary gatekeeper of the home food environment [14,15]. The important role of mothers has been supported by a host of studies revealing mother-child similarities in food intake (see for a review and meta-analysis) [16]. These similarities have also been reported in adolescence [17,18]. However, as youth become more independent of their parents during adolescence, other sources of socialization could become more important during this developmental period. Notably, during adolescence, youth spend increasingly more time with their peers [19,20], making them particularly susceptible to influence from their best friends [21]. Therefore, it is not surprising that food intake similarities between adolescent best friends have been reported as well (see for a review) [22].

Although previous research has shown that both mothers’ and best friends’ food intake are associated with adolescents’ food intake, research on parent and peer influence has developed largely in isolation of one another. Studies that only focused on the role of parents support a pattern in which mother-child similarities are larger for healthy food (e.g., fruit and vegetables) than for unhealthy snacks [17]. Similarities may even be larger when healthy food is obtained from home, with mothers being gatekeepers of the home food environment [23,24]. Studies that have solely assessed the role of friends, generally revealed that similarities among friends are often found for foods typically consumed in peer contexts [25]. Given that these foods are usually unhealthy in nature (e.g., SSBs or snacks) [26,27] and are obtained from outside the home (e.g., at the school canteen, at the snack bar, or at the supermarket) [28], adolescents’ intake is expected to be more similar to their best friends’ food intake when considering unhealthy food obtained from outside the home. The few studies that have addressed the role of parents and friends simultaneously, showed positive associations between adolescents’, parents’, and friends’ intake of SSBs [27] and found that adolescents’ fruit and vegetables intake was positively related to their parents’, but not to their friends’ intake [29].

To broaden our understanding of the relative importance of mothers’ and best friends’ food intake, this study addressed their roles simultaneously while extending most previous research in two ways. First, we included a range of both unhealthy (i.e., SSBs, sweet snacks, and savory snacks) and healthy food (i.e., fruit and vegetables) obtained both from home and from outside the home. As previously discussed by Stok et al. [25], distinguishing between different food types and contexts may help to explain previous, seemingly, conflicting findings regarding the importance of parents and peers. Second, we obtained mothers’ and best friends’ self-reported food intake, in order to not solely rely on adolescents’ perceptions of the food intake of these socializing agents. Previous studies often relied on adolescents to report on their own, and on their parents’ and friends’, food intake behavior, hence potentially overestimating their degree of similarity due to projection [30,31].

In addition to understanding food intake similarities between adolescents and their mothers and best friends, it is critical to better comprehend under which conditions food intake similarities are most pronounced. One of these conditions may be the degree to which adolescents are exposed to their mothers’ and best friends’ food intake. The social learning theory [32] posits that people can adapt to the behavior of others after having observed this particular behavior in others. Illustratively, adolescents may have the opportunity to observe and adapt to their mothers’ food intake when mothers consume food in the presence of their children (e.g., during after-school snacking or during dinner), but may lack this opportunity when mothers consume food in the absence of their children (e.g., at work or when the children are in bed) [33]. Similarly, adolescents may be able to observe and adapt to their best friends’ food intake when best friends consume food in the presence of adolescents (e.g., during school breaks), but not when best friends consume food in the absence of adolescents (e.g., at home). Hence, it was expected that when adolescents are frequently exposed to the food intake of their mothers and best friends, food intake similarities are more pronounced than when adolescents are less frequently exposed to their mothers’ and best friends’ food intake, respectively.

This study had two aims. The first aim was to assess mothers’ and best friends’ food intake in association with adolescents’ intake of unhealthy (i.e., SSBs, sweet snacks, and savory snacks) and healthy food (i.e., fruit and vegetables) obtained from home and from outside the home. It was hypothesized that adolescents show more similarities to their mothers’ food intake when healthy foods obtained from home are considered, while showing more similarities to their best friends’ food intake when unhealthy foods obtained from outside the home are considered. The second aim was to assess the moderating role of adolescents’ exposure to their mothers’ and best friends’ food intake (i.e., mothers and best friends eating in the presence of adolescents) in their food intake similarities, respectively. A larger degree of exposure was hypothesized to magnify food intake similarities between adolescents and their mothers and best friends. When testing the hypotheses, we controlled for adolescents’ age and educational level, as unhealthy food intake seems to be more frequent in older and lower educated youth [34,35,36]. We also controlled for adolescents’ zBMI and gender, as food intake seems to vary by weight status and between males and females [34,36].

## 2. Materials and Methods

### 2.1. Participants

The participants in the present study were part of Wave 1 of the “G(F)OOD together” research project, a longitudinal study on Dutch adolescents’ and their parents’ health behavior. This cohort study includes four waves of data collection, of which the first wave took place in the fall of 2017. Adolescents were recruited through six secondary schools in the South and the East of the Netherlands. A total of 667 adolescents (53% female) from 68 classrooms participated in Wave 1, of which most were born in the Netherlands (98%). All adolescents (*M*_age_ = 12.9 years; *SD*_age_ = 0.7; and age range = 10.0 to 14.8 years) attended secondary education, either in their first year (US Grade 7; *n* = 460) or second year (US Grade 8; *n* = 207). Most adolescents (58%) were in pre-university education (Dutch: havo/vwo or vwo), 8% was in higher general secondary education (Dutch: vmbo-t/havo or havo), and 34% was in pre-vocational education (Dutch: vmbo).

Additionally, parents of the adolescents were also invited to participate in the research project. In total, 396 biological mothers (59% of the adolescent sample) participated at Wave 1. The mean age of the mothers was 44.7 years (*SD*_age_ = 4.2; age range = 29.8 to 57.3 years) and most mothers were born in the Netherlands (97%). Regarding employment, 19% of the mothers was employed full time (i.e., worked 32 h per week or more), 53% were employed part time (i.e., worked less than 32 h per week), 14% were self-employed, and 14% were unemployed (e.g., being a student, stay-at-home mom, or looking for a job). Regarding educational level, mothers obtained either a university degree (11%) or finished higher professional education (40%), secondary vocational education (39%), or primary or secondary education only (10%).

### 2.2. Procedure

The current study was conducted in accordance with the Declaration of Helsinki, and the study procedures were approved by the Ethics Committee Social Sciences of Radboud University, Nijmegen, The Netherlands (ECSW20170805-516). An active parental consent procedure was used, in which a letter describing the four-wave study was mailed to the parents of 1657 adolescents. The two main caregivers were both invited to participate. Parents were asked to return a (paper or digital) consent form indicating whether they agreed to (1) their child’s participation and (2) their own participation in the study. Children were rewarded with a small gift if at least one of their parents’ forms was returned, regardless of whether consent was provided. Parental consent was provided for 718 children, of which 48 were absent on the day of Wave 1 testing and three did not provide consent to participate in the project themselves (final sample: *N* = 667). Moreover, 777 mothers or fathers provided consent to participate in the study themselves, of which 593 took part in Wave 1. Although the two main caregivers were invited to participate in the project, only in 85 cases, both caregivers actually participated. In cases that both caregivers participated, we only included the biological mothers (*n* = 83; excluded: *n* = 5 non-biological mothers; *n* = 80 biological fathers; and *n* = 2 non-biological fathers). Furthermore, in the remaining sample in which only one caregiver participated, biological mothers participated most often (*n* = 352 biological mothers; excluded: *n* = 1 non-biological mother; *n* = 68 biological fathers; and *n* = 2 non-identifiable), resulting in a sample of 435 biological mothers. As in 28 cases adolescents’ data on the main measures were missing, and in 11 cases mothers’ data on the main measures were missing, the final sample consisted of 396 biological mothers. Given that (a) theoretically, previous studies have mainly focused on the important role mothers play in explaining adolescents’ food intake and (b) the number of fathers who participated was low, we did not include reports from fathers in the present study.

Participants were informed that participation was voluntary, that answers would be processed anonymously, and that they could withdraw from the study at any moment. Adolescents completed an online survey at school during one classroom hour (approximately 45 min). In addition, adolescents’ height and weight were measured at school by two trained research assistants, out of sight of classmates. Adolescents’ height was measured to the nearest 0.1 cm with a Seca stadiometer 217, and their weight was measured to the nearest 0.1 kg with a Seca weighing scale 840. Parents completed an online survey at home (approximately 20 min). Both questionnaires were administered through Qualtrics Survey Software. Thirty-four gift vouchers (values: 5 to 50 euros), and three weekend getaways (value: 250 euros) were raffled among the participating families.

### 2.3. Measures

#### 2.3.1. Food Intake 

To assess adolescents’, mothers’, and best friends’ intake of SSBs, sweet snacks, savory snacks, and fruit and vegetables, participants were asked to complete a food frequency questionnaire (FFQ). Specifically, the FFQ assessed participants’ intake of (1) soft drinks (carbonated and non-carbonated drinks with sugar, diet drinks excluded; e.g., cola or lemonade); (2) cake, pastry, and large cookies (e.g., donut or muffin); (3) candy bars (e.g., chocolate-covered bars or confections); (4) chocolate (e.g., chocolate bars or pralines); (5) warm, fried snacks (e.g., sausage roll or pizza slice); (6) fruit (e.g., apple or banana); (7) salad and raw vegetables (e.g., cherry tomatoes or cucumber); and (8) heated vegetables (i.e., cooked, baked, steamed, or otherwise heated; e.g., broccoli or green beans). For each item, participants could indicate their intake on an eight-point scale ranging from ‘0 days a week’ (0) to ‘7 days a week’ (7). Text and pictures were presented to inform participants about which products to consider for each item. Scores for soft drinks (item 1) were used to obtain the measure for SSBs. Scores for cake, candy bars, and chocolate (items 2–4) were summed to assess sweet snacks. Scores for warm, friend snacks (item 5) were used to assess savory snacks. Scores for fruit, salad and raw vegetables, and heated vegetables (items 6–8) were summed to assess fruit and vegetables.

The items measuring sweet and savory snack intake have been selected from a validated Dutch FFQ measuring fat intake [37]. In line with previous studies [18,36], all items assessing the intake of sweet snacks were included. However, some modifications were made with regard to the items assessing savory snack intake [37]. We disregarded two items on “nuts and peanuts” and on “potato chips, pieces of cheese and sausage”, since the Dutch Nutrition Centre considers (low-fat) cheese and (unsalted) nuts to be part of a healthy diet. The items to assess fruit and vegetable intake were also selected from a validated Dutch FFQ [38]. The only item that was disregarded was the item of fruit juice, as the Netherlands Nutrition Centre does not advise to consume fruit juices instead of whole fruits, as it contains less dietary nutrients and fibers.

For each of the eight food items, *adolescents* were asked to indicate how many days per week (0–7) they obtained this particular item in four different contexts: (1) taken or received from home, to eat or to drink at home or to take away; (2) bought at school, such as from the canteen or the vending machine; (3) bought somewhere else, such as in the supermarket, snack bar, or sports club; and (4) received somewhere else, such as at their neighbors’, grandparents’, or friends’ place. In addition to intake of food obtained from home (context 1), a measure representing intake of food obtained outside the home was constructed by summing the responses on contexts 2 to 4. Responses to all four contexts were summed to obtain a measure of adolescents’ total food intake.

For each of the eight food items, *mothers* were asked to indicate how many days per week (0–7) they consumed this particular item (1) in the presence of their child and (2) in the absence of their child. Responses to both situations were summed to obtain a measure of mothers’ total food intake.

To assess *best friends*’ food intake, adolescents were initially asked to nominate their very best friend from their classroom at school. They were allowed to nominate same- and other-gender classmates, but were not allowed to choose themselves. Once a best friend was identified, this individual’s total food intake score was matched to the adolescents’ scores, in order to be used as the best friends’ food intake measure. Of the 667 participating adolescents, 648 nominated an identifiable best friend from their classroom. Of the 648 identifiable nominations, data from 378 best friends could be matched to the adolescents’ food intake scores (57% of the adolescent sample). In the remaining 270 cases, the friend did not participate in Wave 1 of the research project.

#### 2.3.2. Exposure to Mothers’ and Best Friends’ Food Intake

To assess the degree of exposure to *mothers’* food intake, a proportion score of mothers’ food intake in the presence of their child relative to their total food intake was computed. This score could range from 0 to 1, with 0 representing all intake in the absence of her child (‘zero exposure’), and 1 representing all intake in the presence of her child (‘full exposure’). A separate score was obtained for the intake of SSBs, sweet snacks, savory snacks, and fruit and vegetables. To assess the degree of exposure to *best friends’* food intake, adolescents were asked to answer the question “How often do you eat and drink with (name of chosen best friend) during school breaks?” after nominating their best friend. Adolescents could respond to this question on a six-point scale, with response categories ‘never’ (1), ‘once a month or less’ (2), ‘two to three times a month’ (3), ‘one to two times a week’ (4), ‘three to four times a week’ (5), and ‘every school day (5 days a week)’ (6).

#### 2.3.3. Covariates

Adolescents’ *age* in years (rounded to two decimal points) was derived from their date of birth and date of participation and *educational level* was coded as 1 = lower general secondary education, 2 = higher general secondary education, and 3 = pre-university education. To assess adolescents’ *zBMI*, first adolescents’ BMI was computed by dividing their weight in kilograms by their squared height in meters. Subsequently, adolescents’ zBMI was computed by considering the age- and gender-specific growth curves for BMI, based on a Dutch representative sample of 0-to-21-year-olds [39]. Adolescents’ *gender* was dummy coded, with males = 0 and females = 1.

### 2.4. Statistical Analyses

Pearson’s correlation coefficients between adolescents’, mothers’, and best friends’ food intake and the covariates were initially obtained along with descriptive statistics (i.e., *M* (*SD*) or percentages). To address the research questions, a series of linear regression analyses were performed using the *lavaan* package (version 0.6-5) [40] in R (version 3.6-1, Vienna, Austria) [41]. Regressions were performed with adolescents’ intake of the four food types (i.e., SSBs, sweet snacks, savory snacks, and fruit and vegetables) as obtained from the two contexts (i.e., from home and from outside the home) as eight separate outcome measures. Three sets of analyses were performed with these eight different outcomes. In the first set of analyses, the predictors were mothers’ and best friends’ food intake along with the covariates. In the second set of analyses, the two-way interaction terms between mothers’ food intake and adolescents’ exposure to their mothers’ food intake were included as predictors, along with their main effects and the covariates. In the third set of analyses, the predictors were the two-way interaction terms between best friends’ food intake and adolescents’ exposure to their best friends’ food intake, along with their main effects and the covariates. All continuous predictors were centered before creating the interaction terms. Simple slopes analyses were used to interpret statistically significant interactions by plotting the association between adolescents’ and their mothers’ or best friends’ food intake, separately for high (*M* + 1*SD*) and low (*M* − 1*SD*) levels of exposure [42] using the *pequod* package (version 0.0-5) [43] in R.

The *lavaan* package in R was used in order to account for missing values on the food intake variables of mothers (59% available) and best friends (57% available). Moreover, the package was used to deal with potential issues involving the non-normal distributions of the food intake measures. Specifically, full information maximum likelihood (FIML) was used to estimate each regression model for the entire analytic sample and the Huber–White covariance adjustment (MLR) was applied to the standard errors of each parameter estimate [40]. Because of the nested design (i.e., adolescents nested within classrooms and schools), intraclass correlation coefficients (ICCs) were calculated to estimate the strength of clustering. As ICCs were considered low (i.e., <0.05), no adjustment for nesting was made when performing the regressions.

## 3. Results

### 3.1. Descriptive Statistics

Pearson’s correlation coefficients between adolescents’, mothers’, and best friends’ food intake and the covariates are presented in Supplementary Table S1, along with descriptive statistics (*M* (*SD*) or percentages). The correlation coefficients indicate that for all food types, adolescents’ intake of foods obtained *from home* was related to their mothers’ total food intake, and their mothers’ intake in the presence of their child. Mothers’ intake in the absence of their child was only related to adolescents’ intake of food obtained from home in the case of sweet snacks. When considering the associations of mothers’ intake with adolescents’ intake of foods obtained *from outside the home*, the observed pattern was less consistent. Mothers’ total intake was only associated for the intake of SSBs and savory snacks, while mothers’ intake in the presence of their child was only associated for fruit and vegetables, and mothers’ intake in the absence of her child was only associated for SSBs. None of adolescents’ food intake measures were associated with best friends’ food intake, with the exception of the association with adolescents’ intake of savory snacks obtained from home.

### 3.2. Associations with Mothers’ and Best Friends’ Food Intake

The results in Table 1 consistently show that mothers’ food intake was significantly and positively associated with adolescents’ intake of both healthy and unhealthy foods obtained *from home* (SSBs: β = 0.12, *p* = 0.025; sweet snacks: β = 0.17, *p* = 0.004; savory snacks: β = 0.11, *p* = 0.021; and fruit and vegetables: β = 0.24, *p* < 0.001). In contrast, the association of best friends’ with adolescents’ food intake was non-significant across all food types. For foods obtained *from outside the home*, only the positive association between mothers’ and adolescents’ intake of fruit and vegetables was significant (β = 0.10, *p* = 0.049).

### 3.3. The Moderating Role of Exposure to Food Intake

**Figure 1:**
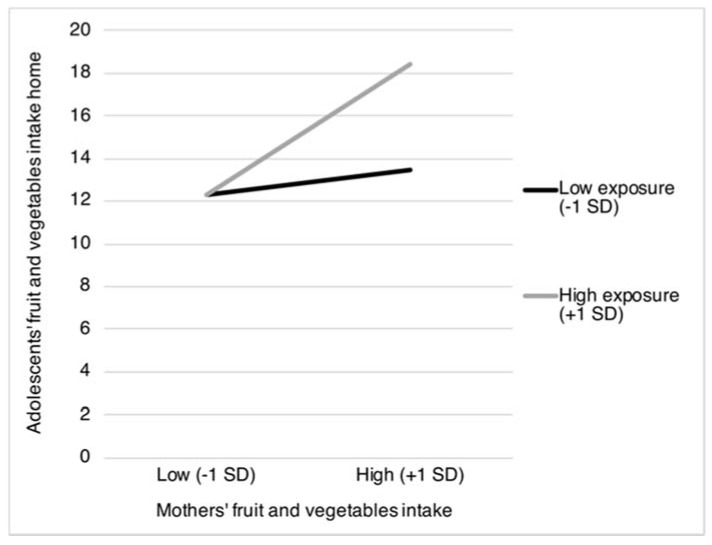
Adolescents’ intake of fruit and vegetables obtained from home as moderated by mothers’ fruit and vegetables intake and the proportion of mothers’ fruit and vegetables intake in the presence of their children. *SD* = Standard Deviation.

**Figure 2:**
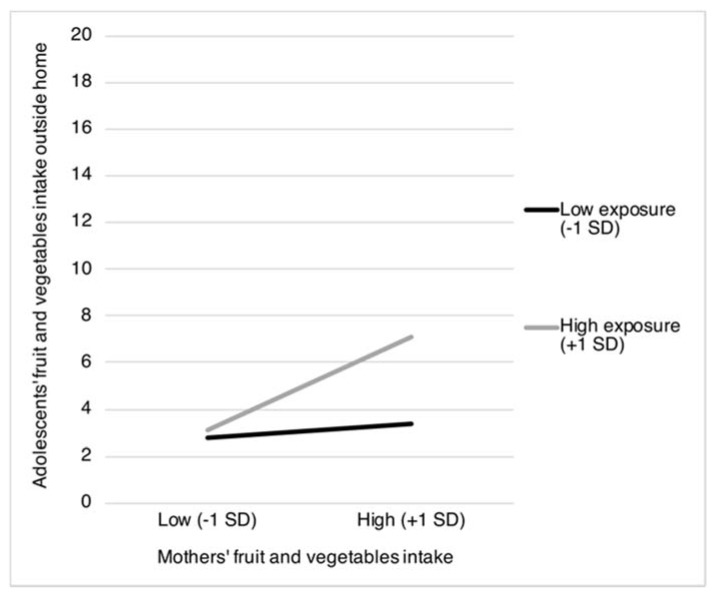
Adolescents’ intake of fruit and vegetables obtained from outside the home as moderated by mothers’ fruit and vegetables intake and the proportion of mothers’ fruit and vegetables intake in the presence of their children. *SD* = Standard Deviation.

The results in Table 2 show two statistically significant interaction terms for exposure to *mothers’ food intake*. Specifically, exposure to mothers’ fruit and vegetables intake was found to moderate the positive associations between mothers’ fruit and vegetables intake and adolescents’ intake of fruit and vegetables obtained both from home (β = 0.27, *p* < 0.001) and from outside the home (β = 0.15, *p* = 0.037). Figure 1 and Figure 2 show that the positive association between mothers’ and adolescents’ fruit and vegetables intake is most pronounced when mothers more frequently consume fruit and vegetables in the presence of their children (from home: *b* = 0.39, *SE* = 0.06, *p* < 0.001; and from outside the home: *b* = 0.25, *SE* = 0.08, *p* = 0.001), compared to when mothers less frequently consume fruit and vegetables in the presence of their children (from home: *b* = 0.03, *SE* = 0.03, *p* = 0.022; and from outside the home: *b* = 0.04, *SE* = 0.04, *p* = 0.358).

**Figure 3:**
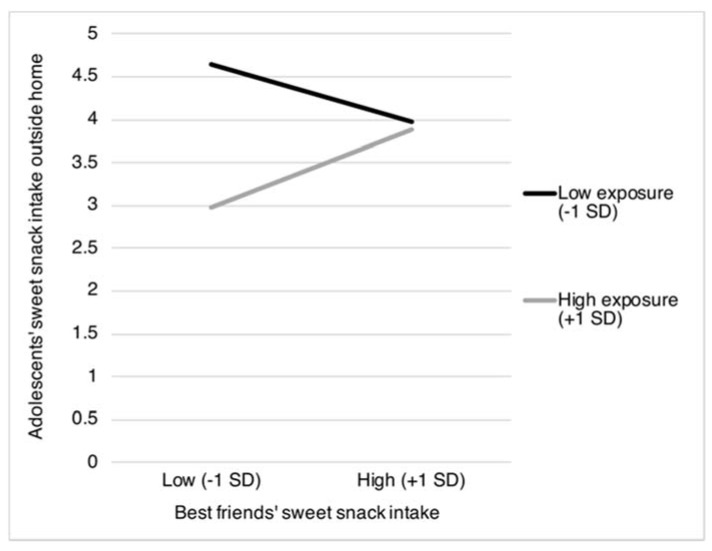
Adolescents’ intake of sweet snacks obtained from outside the home as moderated by best friends’ sweet snack intake and best friends’ frequency of eating and drinking together during school breaks. *SD* = Standard Deviation.

**Figure 4:**
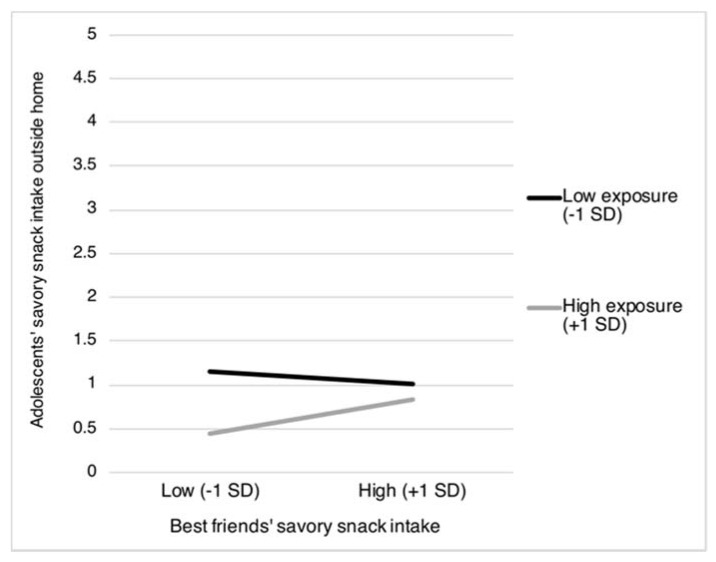
Adolescents’ intake of savory snacks obtained from outside the home as moderated by best friends’ savory snack intake and best friends’ frequency of eating and drinking together during school breaks. *SD* = Standard Deviation.

Moreover, two interaction terms including exposure to *best friends’ food intake* were found to be statistically significant. Specifically, exposure to best friends’ sweet and savory snack intake was found to moderate the association between best friends’ sweet and savory snack intake and adolescents’ intake of sweet and snacks as obtained outside the home (sweet snacks: β = 0.12, *p* = 0.004; and savory snacks: β = 0.15, *p* = 0.024). Figure 3 and Figure 4 reveal that the association between best friends’ sweet and savory snack intake and adolescents’ intake of these snacks obtained from outside the home was most pronounced when best friends more frequently eat and drink together during school breaks (sweet snacks: *b* = 0.06, *SE* = 0.04, *p* = 0.170; and savory snacks: *b* = 0.11, *SE* = 0.05, *p* = 0.019), compared to when best friends less frequently eat and drink together during school breaks (sweet snacks: *b* = −0.04, *SE* = 0.03, *p* = 0.210; and savory snacks: *b* = −0.05, *SE* = 0.58, *p* = 0.328).

## 4. Discussion

The current study aimed to test associations of both mothers’ and best friends’ food intake with adolescents’ intake of unhealthy and healthy food obtained from home and from outside the home, while using self-reports from adolescents, their mothers, and their best friends. Moreover, innovative moderating effects with adolescents’ exposure to their mothers’ and best friends’ food intake were tested, providing insights into the conditions under which food intake similarities are most pronounced. Generally, we found that mothers’ food intake was more often associated with adolescents’ food intake than best friends’ food intake. Additionally, it was revealed that several of these associations were moderated by the degree of exposure. Below, we will further elaborate upon and discuss these findings.

In line with our expectations, we found that mothers’ healthy food intake was related to adolescents’ intake of healthy food. Additionally, we found that mothers’ unhealthy food intake was consistently associated with adolescents’ intake of all types of unhealthy food that were obtained from home. Collectively, these findings underscore the important role mothers play in explaining their children’s food intake, which is in line with a host of studies showing mother-child similarities in food intake [16]. Specifically, our study highlights that mother-child similarities in food intake remain when youth reach adolescence, and that similarities are found for the intake of both healthy [17,29] and unhealthy food [17,18,27]. However, contrary to our expectations, no main associations with best friends’ unhealthy and healthy food intake were found in the current study. Our findings seem inconsistent with findings from studies that report food intake similarities between adolescent best friends (see for a review) [22]. This inconsistency could be explained by the fact that (a) in most previous studies, adolescents were asked to report both on their own and on their friends’ food intake and (b) older samples of adolescents were used. In the current study, we obtained best friends’ self-reported food intake, in order to not solely rely on adolescents’ perceptions of their food intake which could result in potential overestimation of similarity due to projection effects [30,31]. Moreover, our sample mainly included Dutch 12 to 13-year-olds, who had just transitioned from primary to secondary education. Associations with best friends might become stronger when adolescents get older and become more independent from parents. Thus, our study suggests that for early adolescents, parents may still be regarded as the primary socialization agents, at least when it comes to their food intake. Future research should shed light on whether the importance of best friends increases when adolescents develop through middle and late adolescence, while making use of self-reports from adolescent and their socialization agents.

The current study was unique in examining the conditions under which food intake similarities are most pronounced. In line with our expectations, we found that mother-child similarities in the intake of healthy food obtained from home, and even from outside the home, were most pronounced when adolescents were more frequently exposed to their mothers’ healthy food intake (i.e., as reported by mothers). It could be the case that when mothers consume healthy food in the presence of their children (e.g., during lunch or dinner at home), adolescents have the opportunity to observe and adapt to their mothers’ food intake. Mother-child similarities in healthy food intake may thus be explained by social learning effects, including modeling [32,44]. These effects may be more direct within the home environment, but might also be transferred more indirectly through social norms in contexts outside the home environment [7]. Another mechanism that could potentially explain our findings is the clustering of food parenting practices. Illustratively, parents who serve as healthy role models for their children’s food intake, also use other structured food parenting practices (e.g., rules, home food availability, and boundaries) [45,46,47,48] that may explain healthy food intake similarities between mothers and their children. Additionally, since previous studies have shown that food preference and intake are influenced by genetic factors [49,50,51], food intake similarities between mothers and their children could also be due to genetic resemblance. However, given that in this study similarities were more pronounced for adolescents who were more frequently exposed to their mothers’ intake, environmental factors seem to play a bigger role (potentially in addition to genetic factors). Future research should aim to further unravel the specific (combination of) mechanisms that underlie mother-child similarities in food intake. 

As expected, we also found a moderating role for exposure to best friends’ food intake. Specifically, exposure to best friends’ intake of sweet and savory snacks moderated similarities in adolescents’ and their best friends’ intake of these unhealthy products obtained from outside the home. Again, these similarities may be explained by social learning effects, including modeling [32,44]. These social learning effects are likely more direct, as our findings are limited to adolescents’ intake of snacks obtained from outside the home, the context in which adolescents are exposed to their best friends’ food intake (e.g., during lunch breaks at school). Notably, upon inspecting the simple slopes used to interpret the statistically significant interaction, an interesting pattern was observed for both sweet and savory snacks. It was observed that a large degree of exposure to best friends who have a low intake of sweet and savory snacks is related to a strikingly low intake of these unhealthy snacks in adolescents. These findings may suggest that the (more direct) influence from peers may primarily inhibit, rather than augment, adolescents’ unhealthy food intake, in line with previous experimental studies [44,52]. Future research needs to further examine and replicate these seemingly inhibitory best friends’ effects on food intake.

The current study had several strengths and limitations. One notable strength of this study was that the associations with both mothers’ and best friends’ food intake were simultaneously included. Moreover, in the current study, we included a range of both unhealthy and healthy food, obtained both from home and from outside the home, which may help to explain previous seemingly conflicting findings regarding the relative importance of parents and peers. Additionally, adolescents, mothers, and best friends all reported on their own food intake, thereby limiting potentially biased parent and peer reports from adolescents based on projection [30,31]. A limitation of the present study was that although we included information from two important socialization agents (i.e., mothers and best friends), we did not incorporate reports from other potentially important agents (e.g., fathers, siblings, and best friends outside the classroom). Given that studies indicate that these socializing agents may also influence adolescents’ food intake, e.g., [53], future work should aim to simultaneously incorporate these factors, in order to better comprehend adolescents’ complex social world in which they can encounter various (potentially conflicting) sources of socialization. In addition to including information from other important socializing agents, other types of eating behaviors could be considered as well. For instance, examining more disruptive eating behaviors, such as food addiction and loss of control while eating, could be of high relevance in adolescence [54,55,56]. Another limitation of this study is its cross-sectional design, which limits us from drawing conclusions about the direction and causality of the associations found. As previously described [57], parents may very well react on their children’s behavior instead of communicating an example themselves. Furthermore, children likely select friends that show similar food consumption patterns [58]. Future studies could benefit from the use of multiple measurement points over a longer period of time, which could also inform us about the potential development differences in social influence over time. Finally, although our items to measure food intake were based on validated FFQs [37,38], the questionnaire was adapted and should be further validated.

## 5. Conclusions

Taking these limitations into account, the findings of the current study suggest that mothers are relatively more important than best friends in explaining (early) adolescents’ healthy and unhealthy food intake. It could be relevant to make mothers aware of their important role [29,59], both within and outside of the home environment. When best friends consume low levels of sweet and savory snacks, they may have a protective effect on adolescents’ snack intake when they often eat and drink together. It might thus be worthwhile to focus future peer intervention efforts on discouraging intake of unhealthy food, rather than on stimulating intake of healthy food, although more research is needed to corroborate this finding. Additionally, future work needs to further investigate the mechanisms that may underlie these similarities and needs to investigate associations over time and in later developmental periods in youth (e.g., middle and late adolescence). These efforts may help us to better comprehend the relative importance of parents and peers, and may inform the design of effective health promotion interventions that incorporate the social context.

## Figures and Tables

**Table 1:** Standardized regression coefficients testing the associations of mothers’ and best friends’ food intake with adolescents’ food intake (*N* = 667).

	SSBs		Sweet Snacks		Savory Snacks		Fruit and Vegetables	
	Home	Outside Home	Home	Outside Home	Home	Outside Home	Home	Outside Home
Covariates ^a^								
Mothers’ food intake	0.12 *	0.11	0.17 **	0.03	0.11 *	0.09	0.24 ***	0.10*
Best friends’ food intake	0.05	0.06	0.05	−0.01	0.09	0.06	−0.04	−0.04
*R* ^2^	0.04	0.08	0.08	0.04	0.08	0.05	0.09	0.03

Note. * *p* < 0.05. ** *p* < 0.01. *** *p* < 0.001. ^a^ Covariates: Adolescents’ age, educational level, zBMI, and gender. Supplementary Table S2 can be consulted for the regression coefficients.

**Table 2:** Standardized regression coefficients testing the moderating role of adolescents’ exposure to mothers’ and best friends’ food intake (*N* = 667).

		SSBs		Sweet Snacks		Savory Snacks		Fruit and Vegetables	
		Home	Outside Home	Home	Outside Home	Home	Outside Home	Home	Outside Home
Covariates ^a^									
Food intake	M	0.18 **	0.07	0.19 **	0.02	0.05	−0.09	0.43 ***	0.22 **
	BF	0.04	0.05	0.05	0.01	−0.08	0.04	−0.01	−0.03
Exposure to food intake	M	0.06	0.01	0.01	−0.07	0.02	−0.05	0.29 ***	0.19 *
	BF	−0.01	−0.08	−0.02	−0.08	−0.04	−0.15 *	0.11 **	0.06
Food intake *X* Exposure to food intake	M	0.10	−0.09	0.06	−0.01	−0.08	−0.14	0.27***	0.15*
	BF	0.01	0.05	0.09	0.12 **	0.08	0.15 *	0.05	0.04
*R* ^2^	M	0.05	0.08	0.08	0.04	0.07	0.05	0.15	0.05
	BF	0.03	0.08	0.06	0.05	0.07	0.08	0.05	0.03

Note. * *p* < 0.05. ** *p* < 0.01. *** *p* < 0.001. White rows represent coefficients of models testing exposure to mothers’ food intake (M), grey rows represent coefficients of models testing exposure to best friends’ food intake (BF). ^a^ Covariates: Adolescents’ age, educational level, zBMI, and gender. Supplementary Table S3 can be consulted for the regression coefficients.

## Supplementary Materials

The following are available online at https://www.mdpi.com/2072-6643/12/3/786/s1, Table S1: Descriptive Statistics (*M* and *SD*) and Pearson Correlation Coefficients Between the Main Study Variables, Table S2: Standardized Regression Coefficients of Covariates as an Addition to Table 1 in the Main Paper (*N* = 667), Table S3: Standardized Regression Coefficients of Covariates as an Addition to Table 2 in the Main Paper (*N* = 667).
